# Faecal Short-Chain, Long-Chain, and Branched-Chain Fatty Acids as Markers of Different Chronic Inflammatory Enteropathies in Dogs

**DOI:** 10.3390/ani14121825

**Published:** 2024-06-19

**Authors:** Cristina Higueras, Ángel Sainz, Mercedes García-Sancho, Fernando Rodríguez-Franco, Ana I. Rey

**Affiliations:** 1 Department of Animal Production, Animal Nutrition, College of Veterinary Medicine, Complutense University of Madrid, Avda. Puerta de Hierro s/n, 28040 Madrid, Spain; 2 Department of Animal Medicine and Surgery, College of Veterinary Medicine, Complutense University of Madrid, Avda. Puerta de Hierro s/n, 28040 Madrid, Spain; angelehr@ucm.es (Á.S.);

**Keywords:** gut health, desaturase index, elongase index, faecal fatty acid profile, food-responsive enteropathy, immunosuppressant-responsive enteropathy, *Giardia* infection, dogs

## Abstract

**Simple Summary:**

Canine chronic inflammatory enteropathies and *Giardia* infection are gastrointestinal diseases characterised by inflammation of the digestive mucosa. This inflammatory process can induce alterations in components of the intestinal structure, such as lipids, as well as in the homeostasis and intestinal environment. Consequently, we have theorised that variations in the composition of faecal fatty acids could exist between different digestive disorders and could help in their differentiation as well as a more personalised dietary treatment.

**Abstract:**

Chronic inflammatory enteropathies (CIEs) are classified based on treatment trials, and new methods are being sought for earlier differentiation and characterization. *Giardia* infection (GIA) is one of the first differential diagnoses and may be present in CIE-affected dogs. The aim of this study was to evaluate the faecal characteristics and faecal fatty acid profile (short, medium, long, and branched-chain fatty acids) in dogs with food-responsive enteropathy (FRE), immunosuppressant-responsive enteropathy (IRE), and dogs infected with *Giardia* compared to healthy control (HC) animals as a potential non-invasive indicator of intestinal health that helps in the differentiation of CIEs. The C16:1n-7 percentage (*p* = 0.0001) and C16:1n-7/C16:0 ratio (*p* = 0.0001) served to differentiate between HC, FRE, and IRE. IRE dogs presented lower levels of short-chain fatty acids (∑SCFAs) (*p* = 0.0008) and acetic acid (C2) (*p* = 0.0007) compared to the other three groups and lower propionic acid (C3) (*p* = 0.0022) compared to HCs. IRE and GIA presented higher faecal fat content (*p* = 0.0080) and ratio of iso/anteiso branched-chain fatty acids (BCFAs) compared to HC and FRE. Correlations between some fatty acids and desaturation indices with the canine inflammatory bowel disease activity index and faecal characteristics were observed, suggesting that these compounds could play an important role in the pathogenesis of these diseases.

## 1. Introduction

Lipids are one of the major constituents of biological membranes and are organised into a bilayer configuration in which proteins with crucial functions are partially or completely embedded [1]. These phospholipid-based bilayers are important for the preservation of structural integrity, but also allow the regulation of membrane fluidity for signal molecules, metabolites, and ions [2]. Among the fatty acids that are mainly involved in membranes, polyunsaturated fatty acids (PUFAs) have demonstrated a crucial role in gut homeostasis by reducing membrane rigidity and adequately facilitating nutrient absorption [3]. Thus, some changes in the fatty acid profile of the phospholipid membrane or mucus have been associated with inflammatory bowel diseases (IBDs) [2,4].

A gut homeostasis state also contributes to the good functioning of the microbiome, which in turn collaborates in the synthesis of some compounds (short-chain and branched-chain fatty acids) [5]. Short-chain fatty acids (SCFAs) are produced in the colon and derive mostly from anaerobic fermentation of dietary fibres by intestinal bacteria [5]. They are important energy sources for colonocytes and have anti-inflammatory and immunomodulatory effects, increasing the expression of mucins and antimicrobial peptides, promoting crypt differentiation and modulation of tight-junction proteins, and regulating innate and adaptive immune cell generation [6,7]. Other fatty acids, such as branched-chain fatty acids (BCFAs), can also be synthesised by bacteria from branched-chain amino acids (valine, leucine, and isoleucine) [8,9]. Recent investigations have pointed to their potential beneficial effects in developing and maintaining the microbiota and enterocyte health since they are the main component of membrane lipids in numerous bacteria [10,11]. Other fatty acids associated with gut microbiota are odd-chain fatty acids (OCFAs), which can be synthesised from SCFAs, mostly from propionic acid (C3) [12,13].

Previous research on dogs with chronic inflammatory enteropathies (CIEs) reported changes in some of these fatty acids. Thus, some authors found reduced C18:1n-9 [14,15], C18:2n-6 or linoleic acid [15] and n-6 [15,16] in blood samples from sick dogs compared to healthy individuals. Moreover, Crisi et al. [17] observed altered elongase and desaturase indices in the erythrocyte membrane phospholipids as a perturbation of the lipid metabolism. On the contrary, in faecal samples, there are controversial results. Galler et al. [18] found higher levels of linoleic acid and monounsaturated fatty acids (MUFAs) in sick animals, whereas other authors observed lower MUFA proportions as a consequence of a lower desaturase activity [16]. Furthermore, reductions in faecal acetic acid (C2), propionic acid (C3), and ∑SCFAs have also been reported [16,19] in dogs with CIEs. However, most of these studies were carried out to characterize CIEs in general or in specific cases, such as food-responsive enteropathy (FRE) [16] or immunosuppressant-responsive enteropathy (IRE) [18], without there being a comparative study that allows us to evaluate different types of enteropathies from the point of view of the lipid profile of the faeces. Moreover, there are no studies that evaluate to what extent long BCFAs can change in different canine CIEs and if it can help to establish differences between them.

On the other hand, there is no current information on the potential modifications of these compounds in dogs with parasitic disorders such as *Giardia* (GIA), which is one of the first differential diagnoses of CIEs. This parasite is a worldwide intestinal protozoan that infects humans and domestic and wild animals causing gastrointestinal signs [20]. *Giardia* diagnosis is well established using coprological examination, but the risk of false negatives depends on the sensitivity of the diagnostic method [21]. It is well known that *Giardia* depends on exogenous lipids for its growth and differentiation, since it has a limited ability to synthesize its own lipid molecules [22]. As a result, lipids contained in the gut are incorporated into *Giardia* membranes, probably reducing their availability to the host [22].

Since the current classification of CIEs as a group of idiopathic disorders with chronic, persistent, or recurrent gastrointestinal signs [23] relies on treatment response [24], new biomarkers are being sought for earlier differentiation. In consequence, we hypothesize that the general composition and lipid profile of faecal samples in dogs with gastrointestinal chronic disorders could be a non-invasive method for the characterization of these diseases and that the lipid profile may change depending on the severity of the damage to the intestinal structure or homeostasis, and consequently its determination could help in the diagnosis and a more accurate dietary treatment.

Thus, the aims of this study were firstly to evaluate the faecal characteristics and faecal fatty acid profile (short, medium, long, and branched-chain fatty acids) in dogs with chronic digestive diseases (food-responsive enteropathy, immunosuppressant-responsive enteropathy, and *Giardia* infection) compared to healthy individuals, and secondly to look for possible correlations between these fatty acids and the illness severity, as well as its discriminating potential.

## 2. Materials and Methods

All procedures and protocols developed in the present research were approved by the Animal Research Committee of the Complutense Veterinary Medicine Teaching Hospital (CVMTH), (reference 11/2021, approval date: 26 May 2021). Owners of all the dogs accepted their participation through informed consent.

### 2.1. Experimental Design and Animal Signalment

A total of eighty-four dogs were part of this experimental study. Of these, sixty-two were sick dogs that attended the CVMTH Gastroenterology and Endoscopy Service between January 2022 and March 2023. The criteria for inclusion of these dogs were the presence of gastrointestinal signs for at least three weeks consisting of vomiting, diarrhoea, weight loss, and/or anorexia/hyporexia. In addition, twenty-two healthy control (HC) dogs for elective or routine consultations were included in the study. The criteria for inclusion of the latter were a normal physical examination, negative serology for the most common vector-borne diseases in our geographical area (*Ehrlichia* spp., *Anaplasma* spp. and *Leishmania infantum*), negative faecal parasite detection, and the absence of any clinical signs, including digestive signs, or treatments for at least four months. Asymptomatic dogs with chronic diseases were also excluded.

Data on age, sex, fertile status, body weight, body condition score (BCS), muscle condition score (MCS), and canine inflammatory bowel disease activity index (CIBDAI) were collected on the day of the visit (Table 1). The CIBDAI [25] was used for clinical severity evaluation. BCS and MCS were evaluated based on the World Small Animal Veterinary Association (WSAVA) nutritional guidelines [26]. MCS was classified as normal (3), mild (2), moderate (1), or severe (0) muscle loss.

The diagnostic procedure in sick dogs consisted of a complete physical examination, followed by blood sample collection (by jugular or cephalic venipuncture) to rule out other systemic diseases presenting gastrointestinal symptoms (hypoadrenocorticism, pancreatitis, systemic parasites, or infectious diseases), trypsin-like immunoreactivity (TLI) to dismiss exocrine pancreatitis insufficiency, abdominal ultrasound, and a faecal parasitological analysis based on the determination of a modified Telemann method and MIF (merthiolate iodine formaldehyde concentration) technique [27] for the diagnosis of *Giardia* infection. Nine dogs were diagnosed with *Giardia* infection in the present research. Dogs that tested negative were subjected to a dietary trial based on at least two different elimination diets based on novel protein or hydrolysed protein.

Thirty-five dogs were diagnosed with FRE based on a good response to the diet after one month of administration. Dogs that did not respond to the diet change were subjected to endoscopy for biopsy and histological samples [24,28].

Eighteen dogs were diagnosed with IRE based on a poor response to the diet, good response to immunosuppressants, and evidence of an inflammatory process on histological samples. Other causes of gastrointestinal inflammation or systemic diseases were excluded.

Information about the consumed diet was collected for every dog included in the study before starting the dietary trial for diagnosis. The commercial diet’s average composition (percentage) according to the manufacturer was (mean ± standard deviation): humidity, 9.5 ± 0.0; crude protein, 23.8 ± 4.7; crude fat, 14.1 ± 4.2; ash, 6.8 ± 1.3; crude fibre, 2.6 ± 1.4; nitrogen-free extractives, 53.4 ± 8.5; and metabolic energy/1000 g: 4327.2 ± 229.2). A detailed composition of each diet is presented in Supplementary Materials (Table S1).

### 2.2. Faecal Sample Collection

Samples were collected before the start of the dietary trial. Three faecal samples were collected by the owners after defecation on sterile plastic containers for three consecutive days: two were kept under refrigeration until the day of the clinical practice, and the other one was collected the same morning of the visit (these faeces were divided into two different sterile containers). Then, faecal samples were distributed as follows: two refrigerated faecal samples and one fresh were used for parasite detection and the other fresh sample was stored at −20 °C for fatty acid determination. Faecal samples were graded using the Purina^®^ faecal scoring chart [29] on the same day of the visit: from one (very hard and dry sample) to seven (watery sample).

### 2.3. Laboratory Analysis

#### 2.3.1. Total Moisture, Fat Percentage, Total Fatty Acid, and Branched-Chain Fatty Acid Profile in Faecal Samples

Total moisture percentage was calculated by the difference between weighed fresh samples and lyophilised samples [16].

For fatty acid analysis, fat was initially extracted by the addition of a solvent mixture of dichloromethane–methanol (8:2) to lyophilised weighted (0.2 g) samples (Lyoquest, Telstar, Tarrasa, Spain). After homogenisation in a mixer mill (MM400, Retsch technology, Haan, Germany), samples were centrifuged at 10,000 rpm for 10 min. The upper layer containing lipids was collected and placed in 4 mL vials. This process was repeated a second time. After that, the solvents were evaporated in a nitrogen stream and vials were left for 24 h for drying. Total fat percentage (% on a dry matter basis) was calculated by the difference between the 4 mL vial containing extracted fat after evaporation of the solvent and the empty weighed vial with respect to the amount of lyophilised sample weight and multiplied by 100. After that, fatty acid methyl esters (FAMEs) were obtained by heating the lipids at 80 °C for 1 h in the presence of methanol:toluene:H_2_SO_4_ (88:10:2 by volume), as described elsewhere [30]. After esterification, FAMEs were extracted with hexane and separated in a gas chromatograph (HP 6890 Series GC System; Hewlett Packard, Avondale, PA, USA) after direct injection of the sample.

The gas chromatograph was provided with an automatic injector (hold at 170 °C), a flame ionisation detector (hold at 250 °C), and a capillary column (HP-Innowax polyethylene glycol, 30 m × 0.316 mm × 0.25 µm). After injection, the oven temperature was increased to 210 °C at a rate of 3.5 °C/min, then to 250 °C at a rate of 7 °C/min [16]. FAMEs were identified and quantified by comparing their retention times with those of authentic standards (Sigma–Aldrich, Alcobendas, Spain). Results are expressed as grams per 100 g of quantified fatty acids. For branched-chain fatty acid identification, bacterial acid methyl ester (BAME) mix was used (47080-U, Sigma Aldrich, Alcobendas, Spain).

The desaturase indices were calculated as the ratio of C14:1n-5 to C14:0, C16:1n-7 to C16:0, C18:1n-9 to C18:0, C20:1n-9 to C20:0 for Δ-9-desaturase, and C18:3n-6 to C18:2n-6 C18:4n-3 to C18:3n-3 for Δ-6-desaturase. Also, the total Δ-9 and Δ-6-desaturase indices were calculated with the following equations:

Δ-9-desaturase = (C14:1n-5/C14:0) + (C16:1n-7/C16:0) + (C18:1n-9/C18:0) + (C20:1n-9/C20:0)

Δ-6-desaturase = (C18:3n-6/C18:2n-6) + (C18:4n-3/C18:3n-3)

The elongase index was calculated as the ratio of C18:0 to C16:0 and C22:5 to C20:5.

#### 2.3.2. Short-Chain Fatty Acid Analysis in Faecal Samples

Short-chain fatty acid determination was carried out as described elsewhere [16]. One millilitre of distilled water and two glass balls (2 mm Ø) were added to 0.5 g of frozen stool samples. The tubes were homogenised for 5 min at 30 Hz in an MM400 Mixer Mill (Retsch technology, Haan, Germany). Then, separation was conducted via centrifugation (10 min, 10,000 rpm) and the extraction was repeated three times. The superior phase was transferred into a vial, where 20 mM 4-methylvaleric acid solution and 25% phosphoric acid were added to adjust pH at 2–3. Finally, the solution was placed in vials for gas chromatography injection.

Chromatographic analysis was performed using an Agilent 6850N GC system equipped with a flame ionisation detector (FID) (Agilent Technologies, Waldbronn, Germany). A fused-silica capillary column with a free fatty acid phase (DB-FFAP 125-3237, J&W Scientific, Agilent Technologies Inc., Santa Clara, CA, USA) of 30 m × 0.53 mm i.d. coated with a 0.50 µm-thickness film was used. Nitrogen was the carrier gas and maintained at a constant pressure of 15 psi. Initially, the oven temperature was 100 °C for 0.5 min, then raised to 180 °C at 8 °C/min and held for 1 min. After that, it increased to 200 °C at 20 °C/min and finally held at 200 °C for 5 min. The temperatures of the FID and the injection port were 240 °C and 200 °C, respectively. The flow rates of hydrogen, air, and nitrogen as makeup gases were 40, 300, and 30 mL/min, respectively. Data handling was carried out with HP ChemStation Plus software (Rev. B.04.02.98) (Agilent Technologies, Waldbronn, Germany). Pure standards were used for identification and quantification (Sigma-Aldrich, Alcobendas, Spain). An aqueous stock standard solution was prepared for each acid with a concentration of 400 mM for acetic acid, propionic acid, and n-butyric acid; 200 mM for n-valeric acid and i-valeric acid; and 100 mM for i-butyric acid.

### 2.4. Statistical Analysis

Data were analysed following a completely randomised design using the general linear model (GLM) procedure contained in SAS (version 9; SAS Inst. Inc., Cary, NC, USA).

Data are presented as the mean of each group and the standard deviation of the mean (RMSE), together with significance levels (*p*-values). Duncan’s test was used to separate means. The differences between means were considered statistically significant at *p* < 0.05. Pearson correlations (among SCFAs and faecal fatty acids, or CIBDAI and the other variables) were calculated using the Statgraphics-19 program. A linear adjustment between variables was carried out by means of the Statgraphics-19 program (Statgraphics Centurion XIX, X64). Linear discriminant analysis was carried out to discriminate between groups (Statgraphics-19).

## 3. Results

Dog signalment (age, sex, fertile status, breed), body state (weight, BCS, or MCS) and faecal characteristics (Purina^®^ faecal score, faecal moisture and fat content) are presented in Table 1.

Differences concerning age, BCS, and faecal moisture percentage were not statistically affected between groups. On the contrary, HC dogs differed from the other three groups presenting the lowest CIBDAI (*p* = 0.0001) and the highest MCS (*p* = 0.0006). These variables (CIBDAI and MCS) were similar in FRE, IRE, and GIA dogs. Furthermore, body weight (*p* = 0.0059) and Purina^®^ faecal score (*p* = 0.0497) were different in HC dogs when compared to GIA dogs, whereas the FRE and IRE groups presented intermediate values.

The faecal fat content was the stool characteristic that was most affected among the different enteropathies: IRE and GIA dogs had faeces with higher fat percentages than HC and FRE dogs (*p* = 0.008).

Data concerning the total fatty acid profile are presented in Table 2.

HC dogs were differentiated from the other three groups presenting the lowest percentage of C18:0 (*p* = 0.0002), ∑SAT (*p* = 0.0002), and ECFAs (*p* = 0.0001); and the highest percentage of C18:1n-9 (*p* = 0.0007), ∑MUFA (*p* = 0.0008), ratios C16:1n-7/C16:0 (*p* = 0.0001), C18:1n-9/C18:0 (*p* = 0.0003), and Δ-9-desaturase index (*p* = 0.0051). Moreover, this group had the highest percentage of ∑PUFA (*p* = 0.0032) and ∑n-6 (*p* = 0.0055) compared to IRE and GIA dogs, although without statistical differences for FRE dogs.

FRE dogs also presented intermediate percentages of C18:2n-6 (*p* = 0.0128), C20:1n-9 (*p* = 0.0336), C14:1/C14:0 (*p* = 0.0412), C20:1n-9/C20:0 (*p* = 0.0314), and C18:3n-6/C18:2n-6 (*p* = 0.0019) between the HC and IRE groups.

IRE dogs presented the highest faecal percentage of C14:0 (*p* = 0.0122), but the lowest of C16:1n-7 (*p* = 0.0001). This C16:1n-7 was the fatty acid that differed among the HC, FRE, and IRE groups, whereas GIA presented intermediate percentages of C16:1n-7 between HC and FRE dogs (*p* = 0.0001). A similar trend was observed for the ratio C16:1n-7/C16:0, which was different between HC, FRE, and IRE dogs, whereas the GIA group presented similar percentages to FRE (*p* = 0.0001). On the contrary, IRE dogs had higher elongase C18/C16 (*p* = 0.0028) than HC and GIA and presented similar values to FRE dogs. Moreover, IRE dogs presented similar percentages of C14:0, C16:0, C18:0, C18:1n-9, C18:2n-6 and C20:1n-9, ∑SAT, ∑MUFA, ∑PUFA, ∑n-6, Δ-9, C18:3n-6/C18:2n-6 ratio to GIA dogs.

However, GIA dogs differed in the percentage of C18:3n-3 (α-linolenic acid) (*p* = 0.0240) and ∑n-3 (*p* = 0.0020), compared to IRE, HC, and FRE dogs and in C22:5n-3 (*p* = 0.0160) compared to FRE and IRE dogs. Also, the GIA group had a higher percentage of C16:0 (*p* = 0.0079) than the HC and FRE groups.

No statistical differences were observed for the percentages of C14:1, C15:0, C16:1n-9, C17:0, C18:1n-7, C18:3n-6, C18:4n-3, C20:0, C20:2, C20:3n-6, C20:4n-6 (arachidonic acid; AA), C20:5n-3 (eicosapentaenoic acid; EPA), C22:4n-6, total Δ-6-desaturase, elongase C22:5/C20:5, or OCFAs between the experimental groups.

Table 3 presents the long branched-chain fatty acid profile of the experimental groups.

IRE and GIA dogs presented higher ratio iso/anteiso compared to HC and FRE dogs (*p* = 0.0017) and higher iso C17:0 compared to HC dogs, whereas FRE dogs presented intermediate values (*p* = 0.0333). Moreover, the GIA group presented lower levels of anteiso C15:0 (*p* = 0.0054) and total anteiso (*p* = 0.0054) and higher levels of total iso (*p* = 0.0054) compared to HC and FRE dogs. Also, the FRE group showed a higher ∑OCBFA/∑ECBFA ratio when compared to the GIA group (*p* = 0.0122); however, this ratio did not differ between FRE, HC, or IRE dogs. The percentages of iso C16:0 and anteiso C17:0 were not statistically different among the groups of dogs.

Faecal short-chain fatty acid profiles are shown in Table 4. IRE dogs presented lower acetic acid (C2) (*p* = 0.0007) and total ∑SCFAs (*p* = 0.0008) when compared to the other three groups. Propionic acid (C3) of faeces was also different between groups and IRE dogs had lower levels of this SCFA compared to HC dogs, whereas FRE and GIA dogs showed intermediate values (*p* = 0.0022). No differences were observed in the faecal content of isobutyric acid (iC4), butyric acid (C4), isovaleric acid (iC5), or valeric acid (C5) among the experimental groups.

Significant correlations of fatty acid profile, BCFAs, and SCFAs with clinical scores and faecal characteristics are shown in Table 5. CIBDAI and Purina^®^ faecal scores were the variables that correlated to a greater extent with fatty acids.

The greatest number of correlations were observed for C15:0, C16:1n-7, ∑PUFA, ∑n-6 and C16:1n-7/C16:0. CIBDAI correlated positively with faecal ∑SAT (r = 0.36, *p* = 0.0012), C18:0 (r = 0.40, *p* = 0.0002), C22:5n-3 (r = 0.33, *p* = 0.0024), Δ-6-desaturase (r = 0.32, *p* = 0.0040), elongase C18/C16 (r = 0.37, *p* = 0.0006) and elongase C22:5/C20:5 (r = 0.29, *p* = 0.0090). On the contrary, a negative correlation was observed between CIBDAI with C16:1n-7 (r = −0.39, *p* = 0.0003), C18:2n-6 (r = −0.32, *p* = 0.0030), C20:5n-3 (r = −0.22, *p* = 0.0475), ∑PUFA (r = −0.27, *p* = 0.0123), ∑n-6 (r = −0.30, *p* = 0.0062), C16:1n-7/C16:0 (r = −0.45, *p* = 0.0001) and Δ-9-desaturase (r = −0.46, *p* = 0.0001).

C14:1 and C15:0 correlated negatively with Purina^®^ faecal score (r = −0.33, *p* = 0.0025; r = −0.25, *p* = 0.0249) and faecal fat (r = −0.27, *p* = 0.0205; r = −0.23, *p* = 0.0415), respectively. On the contrary, a positive correlation between C15:0 and MCS was found (r = 0.27, *p* = 0.0270). The C16:1n-7 percentage and C16:1n-7/C16:0 ratio correlated positively with MCS (r = 0.25, *p* = 0.0431; r = 0.27, *p* = 0.0329) and negatively with faecal fat (r = −0.34, *p* = 0.0029; r = −0.33, *p* = 0.0041), respectively. A positive correlation was found between C18:0 and Purina^®^ faecal score (r = 0.24, *p* = 0.0298). C18:2n-6 correlated negatively with fat percentage (r = −0.24, *p* = 0.0341). Also, a negative correlation was found between C20:3n-6 and Purina^®^ faecal score (r = −0.28, *p* = 0.0100) and faecal humidity (r = −0.22, *p* = 0.0439). Moreover, C20:5n-3 correlated negatively with Purina^®^ faecal score (r = −0.33, *p* = 0.0030) and C22:5n-3 with MCS (r = −0.26, *p* = 0.0374). Both, ∑PUFA and ∑n-6 correlated negatively with Purina^®^ faecal score (r = −0.26, *p* = 0.0203; r = −0.25, *p* = 0.0230), and fat percentage (r = −0.30, *p* = 0.0095; r = −0.31, *p* = 0.0072), respectively. On the contrary, ∑SAT and elongase C18/C16 correlated positively with Purina^®^ faecal score (r = 0.22, *p* = 0.0486; r = 0.25, *p* = 0.0256).

The BCFA profile also correlated with some faecal characteristics. Thus, anteiso C15:0 correlated negatively with Purina^®^ faecal score (r = −0.23, *p* = 0.0325).

Concerning significant correlations between the SCFAs and faecal characteristics, only negative relationships were found between C2 and faecal fat content (r = −0.27, *p* = 0.0273), as well as between iC4 and moisture content of the stool (r = −0.25, *p* = 0.0352).

**Figure 1:**
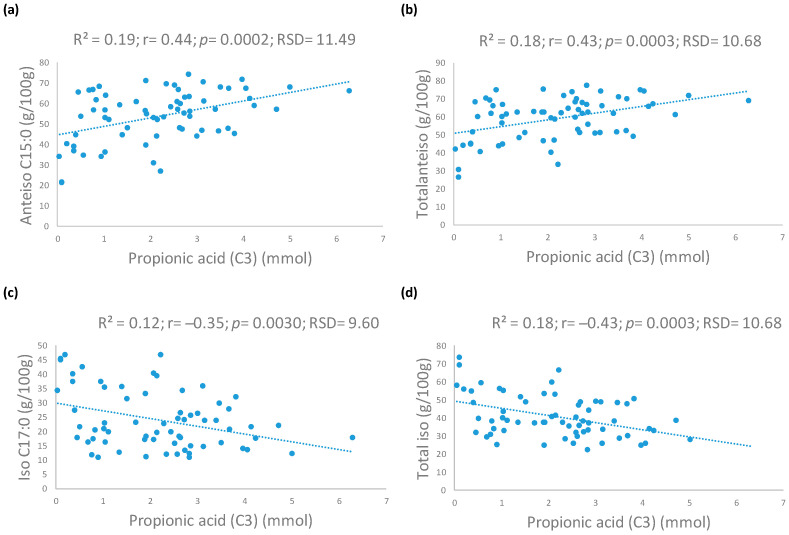
Linear adjustments between (**a**) propionic acid (C3) and anteiso C15:0; (**b**) propionic acid (C3) and total anteiso; (**c**) propionic acid (C3) and iso C17:0; (**d**) propionic acid (C3) and total iso in faecal samples.

The correlations between BCFAs and SCFAs are presented in Figure 1. C3 was the SCFA that correlated the most with BCFAs. A positive correlation was found between C3 and anteiso C15:0 (r = 0.44, *p* = 0.0002) and total anteiso (r = 0.43, *p* = 0.0003), whereas a negative one was found between C3 with iso C17:0 (r = −0.35, *p* = 0.0030) and total iso (r = −0.43, *p* = 0.0003).

**Figure 2:**
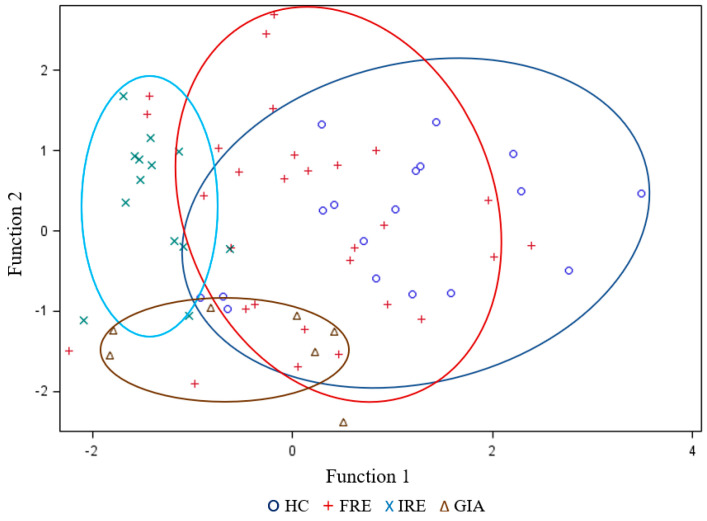
Linear discriminant analysis for faecal samples of healthy control (HC; dark blue) dogs, food-responsive enteropathy (FRE; red) dogs, immunosuppressant-responsive enteropathy (IRE; light blue) dogs and dogs parasitised with *Giardia* (GIA; brown).

Additionally, a discriminant analysis was carried out in order to evaluate the discriminant potential of the studied variables (Figure 2). The most determinant variables that contributed to functions 1 and 2 in the canonical discriminant functions were C16:1n-7/C16:0, C18:1n-9/C18:0, ∑n-3, and C2. Taking into consideration this multivariable analysis, the IRE and GIA groups were the ones that differed the most from the HC and FRE groups, while the FRE and HC groups showed some overlap between them.

The validation results of the discriminant functions (Table 6) showed that 25% of stool samples were not correctly classified, giving a 75% success rate. For the FRE group, 41% were assigned to the correct group; however, 24% were considered GIA, 14% IRE, and 21% HC. The group with the lowest percentage of wrong classifications was GIA (100% correct assignments). However, they had a high percentage of false positives (24% were assigned to FRE, 17% to HC and 8% to IRE). Overall, 92% of faecal samples from IRE dogs were correctly classified, but 8% were wrongly assigned to the GIA group. Finally, 17% of HC samples were considered FRE and 17% GIA.

## 4. Discussion

This is the first study to evaluate changes in faecal characteristics and fatty acid profile among FRE, IRE, GIA and HC dogs. The study was focused on stool samples, since the objective was to search for markers that would establish differences between these chronic gastrointestinal diseases and whose collection would be as little invasive as possible for this purpose.

In the present research, IRE and GIA dogs presented higher fat content in the stools. Taking into account that the fat percentage in the diet was similar in all groups, this result could be attributed to a possible indicator of worse nutrient absorption, probably caused by greater intestinal damage. *Giardia* is a primary alternative diagnosis for CIEs, since it can cause acute or chronic diarrhoea. Despite this, many hosts are still asymptomatic, leading to a debate if the parasite alone is responsible for the gastrointestinal signs or other factors such as dysbiosis, drugs, antimicrobial treatments, infections, and nutritional or environmental changes are implied [31]. However, mechanical mucosal damage caused by *Giardia* infection can lead to fat malabsorption, which in turn can cause intestinal steatosis and increased lipid transit into the distal small intestine and colon [31]. This result was accompanied by worse stool consistency based on Purina^®^ faecal score. On the other hand, the increase in faecal fat in dogs with IRE could be due to a greater disruption of bile acid metabolism resulting from alterations in their transporters or the dysbiosis process [32]. One of the primary functions of bile acids is acting as micelle-forming surfactants that solubilise lipids, facilitating their absorption in the intestine [33]. Alterations in the metabolism of these bile acids have been described in human patients with IBD, especially Crohn’s disease [32], as well as in CIE dogs [18,34], as a consequence of dysbiosis and alterations in the transporters responsible for their reabsorption. Only a very small portion of these acids is not reabsorbed and passes into the colon, where bacteria convert primary bile acids into secondary ones [18]. However, when losses exceed a certain threshold and the compensatory synthetic capacity of the liver is limited, there is a reduction in the intra-duodenal pool of bile acids, limiting micelle formation and thereby resulting in deficient lipid digestion with increased faecal fat content [32]. Fat malabsorption is accompanied by an increase in colonic fluid secretion, with fatty acids significantly involved in this phenomenon [32].

Interestingly, although no significant differences in BCS were observed between groups, sick dogs did present lower MCS compared to HC, with the GIA group being the most affected one, followed by IRE. It has been reported that the profile of circulating fatty acids available for metabolic use could influence MCS values, since fat is the major source of energy for the muscle. Thus, a relationship between muscle mass indices and fatty acid profile has been observed in humans [35], with the polyunsaturated/saturated ratio being the most positively associated with higher muscle mass indices. Furthermore, a direct relationship has been reported between the loss of muscle mass and chronic inflammation, related to a greater synthesis of products involved in the inflammatory process [36]. Polyunsaturated fatty acids play a fundamental role in the development of inflammatory diseases through the synthesis of mediators such as prostanoids, leukotrienes, lipoxins, etc. [37]. The lower proportion of ∑PUFA, mainly ∑n-6, found in faecal samples of IRE and GIA dogs could indicate the higher use of these fatty acids for the synthesis of compounds involved in the inflammation process and therefore a lowered source of energy for the muscle that could lead to greater loss of mass. These results agree with a higher CIBDAI score in sick dogs, mainly IRE and GIA dogs, indicative of a worse clinical status in these animals.

Moreover, sick dogs also displayed alterations in fatty acid metabolism, characterised by higher ∑SAT, lower ∑MUFA and lower Δ-9-desaturase index. These results coincide with previous studies in FRE dogs [16] and CIE dogs [17]. In cases of active human ulcerative colitis, studies have indicated a decrease in the gene expression of Δ-9-desaturase [38], while in mice with colitis, there was a significant inhibition observed in the liver expression of this enzyme [39,40]. However, in the present study, it is interesting to highlight that although IRE dogs did not present significant changes in the desaturase indices (Δ-9) compared to the other groups of sick animals, this group appeared to be the one with the greatest alteration in fatty acid profile. Thus, IRE dogs presented a clear difference in the percentage of C16:1n-7 and ratio of C16:1n-7/C16:0, which were the lowest compared to the other groups. In consequence, this C16:1n-7 could be an interesting fatty acid to consider in the differentiation of IRE dogs. C16:1n-7 has received much attention owing to its anti-inflammatory and metabolic properties, being described as a lipid hormone. The anti-inflammatory character of this fatty acid suggests that its release is a crucial regulatory step for the initiation of pathways aimed at reducing inflammatory damage [41]. Akawaza et al. [42] reported higher serum levels of this fatty acid in humans with Crohn’s disease, and they found a positive correlation with the severity clinical index. However, in the present research, in which faeces samples were evaluated, a negative relationship between C16:1n-7 and CIBDAI score was found. C16:1n-7 may have anti-inflammatory effects on colon inflammation by modulating the IL-6/STAT3 and TNF-α/NF-κB pathways in mouse models [43]. The lower levels of C16:1n-7 in faecal samples in dogs with IRE could in part be explained by a possible lower Δ-9-desaturase activity at the intestinal membrane due to the gut damage [44] or down-regulation by other fatty acids [45,46]. More research is needed to understand changes in this fatty acid in IRE dogs.

On the other hand, IRE dogs presented the highest levels of C18:3n-6/C18:2n-6 (as an indicator of Δ-6-desaturase) in the stool, and this parameter differed from the HC group, but not from the others. This fact, added to a diminution in linoleic acid (C18:2n-6), a main precursor of the n-6 route, suggests that C18:2n-6 is being used to synthesize longer-chain n-6-PUFAs, which are major components of gut cell membranes [47,48], or the synthesis of compounds involved in the inflammatory process [37]. This result agrees with previous studies in the erythrocyte membrane of CIE dogs [17] and humans with Crohn’s disease [49] where the metabolism of linoleic acid by means of the Δ-6-desaturase enzyme is accelerated to a greater extent compared to that of healthy individuals. Thus, lower levels of ∑PUFA and ∑n-6 in faeces (mainly observed in IRE and GIA dogs) were correlated with higher levels of Purina^®^ faecal score and fat percentage, indicators of a worse clinical state. This result aligns with a study where n-6 was negatively correlated with canine chronic enteropathy clinical activity index (CCECAI) scores, although in blood membranes [17]. In addition, the elongase C22:5n-3/C20:5n-3 ratio positively correlated with CIBDAI scores, suggesting that the demand for these fatty acids was higher in animals with more marked intestinal damage. Polyunsaturated fatty acid oxidation products generated have a potent influence on inflammation processes. Thus, oxylipins formed from n-3 PUFAs have more potent anti-inflammatory properties than those formed from n-6 [50]. Therefore, these fatty acids may be consumed as the clinical activity of the disease increases for the repair of intestinal membranes or the synthesis of derivatives involved in the inflammation [37].

Although the GIA group showed similar trends and values for desaturase indices, they differed from the other three groups, presenting the lowest α-linolenic acid (C18:3n-3) and ∑n-3 in the stool. C16:0, C18:0, and C18:1 are the major fatty acids in *Giardia*; however, small amounts of C18:3n-3 have been observed in their composition [51]. It might be possible that GIA dogs presented lower levels of these acids in their faeces to incorporate them into their structure and continue their cycle. Moreover, as suggested in the IRE group, these fatty acids could be used to synthesise pro-resolving mediators during chronic inflammation [37].

Concerning the long BCFAs, these saturated fatty acids are classified as iso (iBCFAs) or anteiso (aiBCFAs) depending on the position of their methyl branch [11]. They have been used as markers of ruminal colonization as one of the main components of ruminal bacteria membranes [52]. In the present research, IRE and GIA dogs had lower anteiso BCFAs than HC and FRE dogs, but higher iso forms. Similar results were observed in the study of Xin et al. [10], where total iso and ratio of iso/anteiso BCFAs were more elevated in the faeces of diarrheic calves compared to healthy controls. However, to the authors’ knowledge, there is no further information, and this study provides for the first time the long BCFA profile in faecal samples of dogs diagnosed with CIEs and *Giardia* infection. Yan et al. [53] showed that anteiso BCFAs presented a more potent inhibitory effect on proinflammatory interleukin 8 production than iso forms in a group of intestinal cells cultivated with BCFAs under lipopolysaccharide stimulation. Both anteiso and iso forms presented anti-inflammatory properties through inhibition of nuclear factor kappaB expression, but anteiso forms reported comparable inhibitory proinflammatory effects to docosahexaenoic acid (DHA) and EPA [53]. Hence, lower anteiso BCFAs in faecal samples of IRE and GIA dogs could be caused by a different bacterial population or a greater utilization of anteiso forms to counteract the inflammatory process that seems to be more severe in these pathologies. This hypothesis could be valid, considering that clinical severity increases from FRE to IRE [54]. Nevertheless, more studies regarding the BCFA profile are needed.

Unlike BCFAs, SCFAs have been more studied to date since they are key prebiotics for maintaining intestinal health, exerting anti-inflammatory, anticancer and immunomodulatory functions in the gut [55]. Recent investigations have reported alterations of SCFAs in dogs with CIEs [16,19] and gastrointestinal diseases such as IBD and colorectal cancer in humans and rodent models [55]. Previous research found lower content of C2, C3, and ∑SCFAs in FRE dogs compared to healthy animals [16]. However, the present research provides for the first time an evaluation of SCFAs between FRE, IRE, and GIA dogs. It is interesting to highlight that IRE differed from the other three groups, presenting the lowest levels of C2, ∑SCFAs, and ∑C2 + C3 + C4 and the lowest levels of C3 compared to HC dogs, but not to FRE or GIA dogs, which presented intermediate values. This result could be attributed to a higher degree of dysbiosis in this IRE group compared to the others, in which reductions of the Bacteroidetes and Firmicutes phyla (SCFAs producers) have been observed [56]. However, only a differential abundance of bacteria belonging to the phylum Proteobacteria was found between samples of intestinal mucosal microbiota in FRE and IRE dogs, whereas no differences in the overall species richness were observed [57]. Although it seems that both groups have similar dysbiotic microbiomes, studies evaluating both lipid profiles and microbiome differences in faecal samples between groups are needed to prove or disprove this hypothesis. In the present research, however, lower levels of these microbial metabolites in IRE dogs could suggest a greater disruption in their bacterial producers. This could lead to a new reclassification of this category in the future [58]. On the other hand, some research points out that dogs with giardiasis do not experience significant dysbiosis [59]. *Giardia* has an impact on gut microbiota that is enriched in protective taxa against gut inflammation and depleted in lipid-producing taxa, which limits *Giardia* infection and reduces host inflammation [31,59]. This supports the idea that there may be a delicate relationship between this parasite and the gut microbiota.

Another result to comment on from the present research is related to OCFAs. C15:0 and C17:0 are OCFAs that can be endogenously synthesised from propionic acid (C3) [12]. In the present research, these OCFAs were not significantly differently affected between the different groups, despite the fact that C15:0 was related to better faecal characteristics (lower Purina^®^ score and faecal fat content). This result does not coincide with previous research in which healthy dogs had higher faecal C15:0 when compared to sick animals [16]. The authors suggested that higher levels of this fatty acid could be attributed to greater microbiome activity [60,61]. However, discrepancies found in the current investigation could be due to the fact that this fatty acid can also be obtained from the diet, which was not exactly the same in both experiments. Jenkins et al. [13] found that circulating C15:0 was linearly related to dietary C15:0 intake, and hardly any contribution was observed on C15:0/C17:0 levels by the gut microbiota.

Concerning the BCFAs, positive correlations were found between C3 and anteiso C15:0 and total anteiso, whereas the relationship was negative with iso C17:0, total iso, and ratio iso/anteiso. These BCFAs are predominant in certain bacteria [62] and are mainly synthesised from malonyl-CoA esters that carry methyl branches, which can be obtained from the degradation of branched amino acids [63]. Thus, although C3 and iso and anteiso BCFAs come from different substrates, both are related to microbial action. The IRE group, which had the significantly lowest C3 content, also presented significantly lower anteiso BCFAs but higher iso forms compared to healthy individuals. Moreover, similar relationships have been found between these BCFAs and clinical parameters such as Purina^®^ faecal score, suggesting that anteiso forms in faecal samples are associated with a better clinical state compared to iso. Since the function of these fatty acids is largely unknown in mammals, more studies are necessary to understand these changes.

In addition, CIBDAI correlations demonstrated that sick dogs presented an altered metabolic state with lower desaturase activity (Δ-9-desaturase, responsible for the conversion of C16:1n-7/C16:0 and C18:1n-9/C18:0) and thus lower C16:1n-7, and higher elongase activity and Δ-6-desaturase, responsible for a faster metabolisation of linoleic acid and α-linolenic acid for long-PUFA production.

Finally, the discriminant analysis of the lipid profile to differentiate different diseases would indicate that the IRE and GIA groups were the ones best differentiated from the rest of the groups considering the variables C16:1n-7/C16:0, C18:1n-9/C18:0, ∑n-3, and C2. FRE dogs differentiated the worst from the rest of the groups. However, only 13.79% of false negatives from FRE dogs were confused with IRE dogs. Therefore, the lipid profile and specifically the desaturase index seem to be interesting indicators of the state of severity and could be a variable to consider to establish more precise diagnosis and dietary treatments between FRE and IRE dogs.

## 5. Conclusions

In conclusion, the faecal fatty acid profile showed alterations between dogs affected by different chronic inflammatory enteropathies and *Giardia* infection compared to healthy dogs. Among sick animals, IRE dogs showed clear differences in the faecal percentage of C16:1n-7 and ratio of C16:1n-7/C16:0 and also differed in the content of C2 from the other groups and in C3 from HC dogs. However, FRE dogs did not present such marked differences in SCFAs or BCFAs. Future studies combined with microbiota analysis or systemic changes will allow for a better understanding of the use of lipid compounds and how to utilize them to improve earlier diagnosis and dietary treatment response.

## Figures and Tables

**Table 1:** Signalment, epidemiological data, and clinical scores of healthy control (HC) dogs, food-responsive enteropathy (FRE) dogs, immunosuppressant-responsive enteropathy (IRE) dogs, and dogs parasitised with *Giardia* (GIA).

Variables	HC (*n* = 22)		FRE (*n* = 35)		IRE (*n* = 18)		GIA (*n* = 9)		RMSE ^4^	*p*-Value
			
Age (months; mean[range])	60.23	[7–144]	57.31	[8–144]	76.17	[9–136]	64.22	[4–144]	42.066	0.4786
Sex (male/female)	9/13		19/16		6/12		7/2		0.494	0.1286
Fertile status (neutered/entire)	12/10		20/15		12/6		4/5		0.503	0.7351
Breed (pure/mixed)	15/7		30/5		13/5		8/1		0.411	0.3344
Weight (kg); (median [range])	19.40 ^a^	[7.5–55]	10.60 ^ba^	[2.6–45.5]	7.32 ^ba^	[1.19–36.2]	12.60 ^b^	[2.7–26.3]	12.358	0.0059
CIBDAI ^1^ (median [range])	0.00 ^b^	[0]	5.00 ^a^	[1–8]	6.00 ^a^	[1–9]	6.00 ^a^	[1–10]	2.004	0.0001
BCS ^2^ (1–9); (median [range])	5.00	[3–6]	5.00	[2–8]	4.00	[2–8]	3.00	[3–6]	1.467	0.2625
MCS ^3^ (0–3); (median [range])	3.00 ^a^	[3]	3.00 ^b^	[1–3]	2.50 ^b^	[1–3]	2.00 ^b^	[1–3]	0.538	0.0006
***Faecal characteristics***										
Purina^®^ faecal score (1–7); (mean [range])	2.50 ^b^	[1–5]	3.14 ^ba^	[1–7]	3.56 ^ba^	[1–7]	4.11 ^a^	[2–6]	1.586	0.0497
Moisture (%); (mean [range])	67.53	[57.05–75.22]	68.40	[55.63–90.73]	71.15	[53.46–91.14]	71.51	[58.24–84.90]	7.651	0.3391
Fat (%); (mean [range])	6.53 ^b^	[4.25–11.93]	6.76 ^b^	[2.97–15.05]	10.02 ^a^	[3.40–23.39]	10.40 ^a^	[3.85–19.48]	3.839	0.0080

^1^ CIBDAI: canine inflammatory bowel disease activity index; ^2^ BCS: body condition score; ^3^ MCS: muscle condition score; ^4^ RMSE: root mean squared error; *p*-value significant when <0.05. Values with different superscript letters (^a,b^) statistically significant.

**Table 2:** Faecal fatty acid profile (g per 100 g) of healthy control (HC) dogs, food-responsive enteropathy (FRE) dogs, immunosuppressant-responsive enteropathy (IRE) dogs and dogs parasitised with *Giardia* (GIA).

%	HC (*n* = 22)		FRE (*n* = 35)		IRE (*n* = 18)		GIA (*n* = 9)		RMSE ^10^	*p*-Value
			
C14:0	1.231	^b^	1.393	^b^	1.773	^a^	1.503	^ba^	0.513	0.0122
C14:1	0.319		0.261		0.300		0.250		0.107	0.1591
C15:0	0.716		0.641		0.647		0.617		0.233	0.6041
C16:0	25.158	^b^	26.852	^b^	28.088	^ba^	30.564	^a^	4.078	0.0079
C16:1n-9	0.238		0.229		0.264		0.259		0.090	0.5417
C16:1n-7	1.968	^a^	1.571	^b^	1.054	^c^	1.834	^ba^	0.475	0.0001
C17:0	0.526		0.533		0.581		0.547		0.156	0.6829
C18:0	15.613	^b^	21.654	^a^	25.164	^a^	20.821	^a^	6.599	0.0002
C18:1n-9	25.155	^a^	19.165	^b^	17.640	^b^	19.338	^b^	6.111	0.0007
C18:1n-7	4.726		5.221		5.299		6.145		1.845	0.2838
C18:2n-6	16.343	^a^	14.099	^ba^	11.448	^b^	11.490	^b^	5.053	0.0128
C18:3n-6	0.098		0.114		0.126		0.109		0.040	0.1718
C18:3n-3	1.248	^a^	1.072	^a^	1.087	^a^	0.754	^b^	0.399	0.0240
C18:4n-3	0.061		0.057		0.063		0.048		0.021	0.3244
C20:0	0.686		0.764		0.700		0.890		0.255	0.1938
C20:1n-9	0.550	^a^	0.513	^ba^	0.412	^b^	0.503	^ba^	0.149	0.0336
C20:2	0.250		0.248		0.265		0.277		0.087	0.7631
C20:3n-6	1.540		1.666		1.467		1.136		0.582	0.1070
C20:4n-6	1.658		1.903		1.684		1.400		0.638	0.1562
C20:5n-3	0.577		0.437		0.440		0.489		0.167	0.1119
C22:4n-6	0.361		0.325		0.319		0.272		0.121	0.3011
C22:5n-3	0.978	^ba^	1.282	^a^	1.180	^a^	0.755	^b^	0.491	0.0160
∑SAT ^1^	43.930	^b^	51.837	^a^	56.953	^a^	54.940	^a^	9.143	0.0002
∑MUFA ^2^	32.956	^a^	26.961	^b^	24.969	^b^	28.329	^b^	6.401	0.0008
∑PUFA ^3^	22.865	^a^	20.954	^ba^	17.813	^bc^	16.453	^c^	5.177	0.0032
∑n-6 ^4^	20.000	^a^	18.107	^ba^	15.043	^bc^	14.408	^c^	5.046	0.0055
∑n-3 ^5^	2.864	^a^	2.847	^a^	2.770	^a^	2.046	^b^	0.607	0.0020
C14:1n-5/C14:0	0.259	^a^	0.188	^ba^	0.170	^b^	0.166	^b^	0.102	0.0412
C16:1n-7/C16:0	0.078	^a^	0.059	^b^	0.038	^c^	0.060	^b^	0.018	0.0001
C18:1n-9/C18:0	1.611	^a^	0.885	^b^	0.701	^b^	0.929	^b^	0.780	0.0003
C20:1n-9/C20:0	0.802	^a^	0.672	^ba^	0.589	^b^	0.566	^b^	0.287	0.0314
C18:3n-6/C18:2n-6	0.006	^b^	0.008	^ba^	0.011	^a^	0.010	^ba^	0.005	0.0019
C18:4n-3/C18:3n-3	0.049		0.053		0.058		0.063		0.033	0.6247
Δ-9-desaturase ^6^	2.750	^a^	1.803	^b^	1.497	^b^	1.721	^b^	0.825	0.0001
Δ-6-desaturase ^7^	0.055		0.061		0.069		0.073		0.036	0.3470
Elongase C18/C16	0.621	^c^	0.806	^ba^	0.896	^a^	0.681	^bc^	0.236	0.0028
Elongase C22:5/C20:5	1.695		2.936		2.679		1.543		2.949	0.0943
OCFAs ^8^	1.241		1.174		1.229		1.163		0.305	0.8098
ECFAs ^9^	42.689	^b^	50.663	^a^	55.724	^a^	53.777	^a^	9.122	0.0001

^1^ ∑SAT: sum of total saturated fatty acids; ^2^ ∑MUFA: sum of total monounsaturated fatty acids; ^3^ ∑PUFA: sum of total polyunsaturated fatty acids; ^4^ ∑n-6: sum of total n-6 fatty acids; ^5^ ∑n-3: sum of total n-3 fatty acids; ^6^ Δ-9-desaturase = (C14:1n-5/C14:0) + (C16:1n-7/C16:0) + (C18:1n-9/C18:0) + (C20:1n-9/C20:0); ^7^ Δ-6-desaturase = (C18:3n-6/C18:2n-6) + (C18:4n-3/C18:3n-3); ^8^ OCFAs: odd-chain fatty acids; ^9^ ECFAs: even-chain fatty acids; ^10^ RMSE: root mean squared error; *p*-value was significant when <0.05. Values with different superscripts (^a,b,c^) are statistically significant.

**Table 3:** Faecal long branched-chain fatty acid profile (g per 100 g) of healthy control (HC) dogs, food-responsive enteropathy (FRE) dogs, immunosuppressant-responsive enteropathy (IRE) dogs and dogs parasitised with *Giardia* (GIA).

Variable	HC (*n* = 22)		FRE (*n* = 35)		IRE (*n* = 18)		GIA (*n* = 9)		RMSE ^3^	*p*-Value
			
Iso C15:0	11.57	^b^	11.21	^b^	13.41	^ba^	14.38	^a^	3.473	0.0313
Anteiso C15:0	57.87	^a^	54.76	^ba^	47.80	^bc^	43.09	^c^	12.121	0.0054
Iso C16:0	4.92		4.30		4.80		6.05		1.755	0.0651
Iso C17:0	20.40	^b^	24.97	^ba^	28.51	^a^	30.26	^a^	10.119	0.0333
Anteiso C17:0	5.24		4.76		5.48		6.21		1.983	0.2195
Total iso	36.89	^c^	40.49	^bc^	46.72	^ba^	50.69	^a^	11.291	0.0054
Total anteiso	63.11	^a^	59.51	^ba^	53.28	^bc^	49.31	^c^	11.291	0.0054
Iso/anteiso	0.61	^b^	0.74	^b^	1.05	^a^	1.15	^a^	0.433	0.0017
∑OCBFAs ^1^/∑ECBFAs ^2^	20.55	^ba^	27.08	^a^	22.95	^ba^	17.19	^b^	9.360	0.0122

^1^ ∑OCBFAs: odd-chain branched fatty acids (iso C15:0 + anteiso C15:0 + iso C17:0 + anteiso C17:0); ^2^ ∑ECBFAs: even-chain branched fatty acids (iso C16:0); ^3^ RMSE: root mean squared error; *p*-value was significant when <0.05. Values with different superscripts (^a,b,c^) are statistically significant.

**Table 4:** Faecal short-chain fatty acid (SCFAs) profile (mM) of healthy control (HC) dogs, food-responsive enteropathy (FRE) dogs, immunosuppressant-responsive enteropathy (IRE) dogs and dogs parasitised with *Giardia* (GIA).

Variable	HC (*n* = 22)		FRE (*n* = 35)		IRE (*n* = 18)		GIA (*n* = 9)		RMSE ^7^	*p*-Value

C2 ^1^	3.196	^a^	3.113	^a^	1.630	^b^	2.742	^a^	1.134	0.0007
C3 ^2^	3.019	^a^	2.046	^ba^	1.326	^b^	2.111	^ba^	1.222	0.0022
iC4 ^3^	0.140		0.154		0.090		0.122		0.108	0.3451
C4 ^4^	1.319		1.131		0.834		1.163		0.763	0.3577
iC5 ^5^	0.259		0.312		0.184		0.244		0.205	0.2910
C5 ^6^	0.141		0.119		0.058		0.070		0.197	0.6709
Total SCFAs	8.075	^a^	6.850	^a^	4.111	^b^	6.451	^a^	2.632	0.0008
∑C2 + C3 + C4	7.535	^a^	6.291	^a^	3.791	^b^	6.016	^a^	2.452	0.0008
∑iC4 + iC5	0.399		0.465		0.274		0.365		0.309	0.3045

^1^ C2: acetic acid; ^2^ C3: propionic acid; ^3^ iC4: isobutyric acid; ^4^ C4: butyric acid; ^5^ iC5: isovaleric acid; ^6^ C5: valeric acid; ^7^ RMSE: root mean squared error; *p*-value was significant when <0.05. Values with different superscripts (^a,b^) are statistically significant.

**Table 5:** Significant correlation coefficients between faecal fatty acids (%), branched-chain (%), and short-chain fatty acids (mM) and canine inflammatory bowel disease activity index (CIBDAI), muscle condition score (MCS) or faecal characteristics (Purina^®^ faecal score, fat, moisture contents).

Variable	CIBDAI		MCS		Purina^®^ Faecal Score		Faecal Moisture (%)		Faecal Fat (%)	
**Faecal fatty acids**										
C14:1	–0.18		0.05		–0.33	^b^	–0.14		–0.27	^a^
C15:0	–0.11		0.27	^a^	–0.25	^a^	–0.22		–0.23	^a^
C16:1n-7	–0.39	^b^	0.25	^a^	–0.08		0.08		–0.34	^b^
C18:0	0.40	^b^	–0.14		0.24	^a^	0.18		0.16	
C18:2n-6	–0.32	^b^	0.11		–0.18		–0.14		–0.24	^a^
C20:3n-6	0.08		0.08		–0.28	^b^	–0.22	^a^	–0.21	
C20:4n-6	0.13		0.00		–0.27	^a^	–0.12		–0.21	
C20:5n-3	–0.22	^a^	0.14		–0.33	^b^	–0.05		–0.02	
C22:5n-3	0.33	^b^	–0.26	^a^	–0.01		–0.07		0.22	
∑SAT ^1^	0.36	^b^	–0.13		0.22	^a^	0.16		0.15	
∑PUFA ^2^	–0.28	^a^	0.10		–0.26	^a^	–0.18		–0.30	^b^
∑n-6 ^3^	–0.30	^b^	0.12		–0.25	^a^	–0.18		–0.31	^b^
C16:1n-7/C16:0	–0.46	^b^	0.27	^a^	–0.20		–0.04		–0.33	^b^
Δ-9-desaturase ^4^	–0.47	^b^	0.21		–0.12		–0.08		–0.09	
Δ-6-desaturase ^5^	0.32	^b^	–0.10		0.11		0.07		–0.14	
Elongase C18/C16	0.38	^b^	–0.16		0.25	^a^	0.18		0.13	
Elongase C22:5/C20:5	0.29	^b^	–0.24		0.22		0.07		0.15	
**Branched-chain fatty acids**										
Anteiso C15:0	–0.04		0.12		–0.23	^a^	–0.20		–0.12	
**Short-chain fatty acids**										
C2 ^6^	–0.20		0.10		–0.11		–0.01		–0.27	^a^
iC4 ^7^	0.06		–0.04		–0.16		–0.25	^a^	–0.04	

^1^ ∑SAT: sum of total saturated fatty acids; ^2^ ∑PUFA: sum of total polyunsaturated fatty acids; ^3^ ∑n-6: sum of total n-6 fatty acids; ^4^ Δ-9-desaturase = (C14:1n-5/C14:0) + (C16:1n-7/C16:0) + (C18:1n-9/C18:0) + (C20:1n-9/C20:0); ^5^ Δ-6-desaturase = (C18:3n-6/C18:2n-6) + (C18:4n-3/C18:3n-3); ^6^ C2: acetic acid; ^7^ iC4: isobutyric acid. ^a^ Significant at <0.05 probability level; ^b^ significant at <0.01 probability level.

**Table 6:** Classification of healthy control (HC) dogs, food-responsive enteropathy (FRE) dogs, immunosuppressant-responsive enteropathy (IRE) dogs, and dogs parasitised with *Giardia* (GIA) (% assigned correctly) according to discriminant functions (cross-validation).

	FRE	GIA	IRE	HC	TOTAL (%)
FRE	41.38	24.14	13.79	20.69	100
GIA	0	100	0	0	100
IRE	0	7.69	92.31	0	100
HC	16.67	16.67	0	66.67	100

## Data Availability

Data are contained within the article.

## Supplementary Materials

The following supporting information can be downloaded at https://www.mdpi.com/article/10.3390/ani14121825/s1. Table S1. General composition of diets of healthy control dogs (HC), food-responsive enteropathy dogs (FRE), immunosuppressant-responsive enteropathy dogs (IRE) and dogs parasitized with Giardia (GIA).
