# Supplementary material for: Faecal Short-Chain, Long-Chain, and Branched-Chain Fatty Acids as Markers of Different Chronic Inflammatory Enteropathies in Dogs
Source: Animals (Basel). 2024 Jun 19;14(12):1825. doi: 10.3390/ani14121825 (PMC11201139; doi:10.3390/ani14121825)
Supplement: Supplementary file 1 [file animals-14-01825-s001.zip › animals-3023747-supplementary.pdf]

animals

MDPI

Article

Faecal Short-Chain, Long-Chain, and Branched-Chain Fatty Acids as Markers of Different Chronic Inflammatory Enteropathies in Dogs

Cristina Higueras 1, Ángel Sainz 2, Mercedes García-Sancho 2, Fernando Rodríguez-Franco 2 and Ana I. Rey 1,*

1 Department of Animal Production, Animal Nutrition, College of Veterinary Medicine, Complutense University of Madrid, Avda. Puerta de Hierro s/n, 28040 Madrid, Spain; crhiguer@ucm.es

2 Department of Animal Medicine and Surgery, College of Veterinary Medicine, Complutense University of Madrid, Avda. Puerta de Hierro s/n, 28040 Madrid, Spain; angelehr@ucm.es (Á.S.); mercgarc@ucm.es (M.G.-S.); ferdiges@ucm.es (F.R.-F.)

*Correspondence: anarey@ucm.es

Table S1. General composition of diets of healthy control dogs (HC), food-responsive enteropathy dogs (FRE), immunosuppressant-responsive enteropathy dogs (IRE) and dogs parasitized with Giardia (GIA).

	**HC (*n*=22)**	**FRE (*n*=35)**	**IRE (*n*=18)**	**GIA (*n*=9)**
Crude protein (%; mean ± SD)	23.0 ± 4.6	23.7 ± 4.5	23.7 ± 5.0	22.7 ± 4.9
Crude fat (%; mean ± SD)	13.7 ± 4.1	14.8 ± 4.3	13.4 ± 4.2	13.5 ± 4.4
Crude fibre (%; mean ± SD)	2.8 ± 0.7	2.8 ± 1.8	2.1 ± 1.6	2.5 ± 1.0
Crude ash (%; mean ± SD)	7.6 ± 1.2	6.7 ± 0.8	5.8 ± 1.0	6.2 ± 2.0
Nitrogen-Free Extractives (%; mean ± SD)	52.6 ± 8.6	51.8 ± 7.9	54.8 ± 8.8	54.9 ± 8.7
Metabolic Energy (kcal/1000 grams) (mean ± SD)	4270.0 ± 230.9	4362.0 ± 236.8	4355.0 ± 190.4	4324.7 ± 259.0

SD: standard deviation
